# Postoperative atrial arrhythmias after bilateral lung transplantation with intraoperative V-A extracorporeal membrane oxygenation: a single-center experience

**DOI:** 10.3389/frtra.2025.1601228

**Published:** 2025-06-30

**Authors:** Annalisa Boscolo, Nicolò Sella, Francesco Zarantonello, Raimondo Pittorru, Giulia Mormando, Carlo Alberto Bertoncello, Elena Curmaci, Roberta Ceccato, Valentina Fincati, Paola Masetti Zannini, Angela Bianco, Giordana Coniglio, Elisa Pistollato, Alessandro Zambianchi, Mustaj Sindi, Sabrina Congedi, Gabriella Roca, Arianna Peralta, Luisa Muraro, Giorgia Pacchiarini, Federico Migliore, Manuel De Lazzari, Tommaso Pettenuzzo, Federico Rea, Martina Perazzolo Marra

**Affiliations:** ^1^ Section of Anaesthesiology and Intensive Care, Department of Medicine (DIMED), University of Padua, Padova, Italy; ^2^ Institute of Anesthesia and Intensive Care, Padua University Hospital, Padova, Italy; ^3^ Department of Cardiac, Thoracic, Vascular Sciences and Public Health, University of Padua, Padova, Italy; ^4^ Department of Medicine (DIMED), University of Padua, Padova, Italy

**Keywords:** arrhythmias, lung transplantation, extracorporeal membrane oxygenation, atrial fibillation, transplantation

## Abstract

**Introduction:**

Lung transplantation (LT) is the standard treatment for end-stage chronic respiratory failure that does not respond to other therapies. Advances in surgical techniques and perioperative care have improved survival rates. However, postoperative complications, particularly atrial arrhythmias (AA) remain clinically significant. Although AAs are frequently observed in the early postoperative period, data regarding their incidence and impact on outcomes are scarce. This observational study aims to: (i) assess the incidence of new-onset postoperative AA within one month of bilateral LT; (ii) evaluate their impact on short- and mid-term outcomes; and iii) identify potential predictors.

**Materials and methods:**

We retrospectively reviewed all consecutive bilateral LT recipients admitted to the Intensive Care Unit (ICU) of the University Hospital of Padua between October 2021 and December 2023. Clinical variables, perioperative right heart catheterization data, and echocardiographic measurements were collected.

**Results:**

A total of 85 LT recipients were enrolled. Postoperative AA occurred in 27 patients (32%), with atrial fibrillation emerging as the most common arrhythmia (55.6%). The remaining 58 (68%) patients did not develop any arrhythmic disorder. Many AA patients (22, 81.5%) required treatment with antiarrhythmic drugs or electrical cardioversion. Compared to the control group, AA patients were older (*p*-value 0.002) and usually affected by coronary heart disease (18.5% vs. 5.2%, *p*-value 0.05) and obstructive respiratory disease (55.5% vs. 27.7%, *p*-value 0.004). AA patients more frequently experienced difficult weaning from mechanical ventilation, a higher incidence of postoperative V-A ECMO, more frequent anastomotic complications, and longer ICU stays, as compared to controls. Multivariate analysis identified older age (OR 1.11, 95% CI 1.01–1.25, *p*-value 0.047) and higher postoperative dobutamine dosage (OR 2.25, 95% CI 1.15–5.01, *p*-value 0.026) as the only significant predictors of new-onset AA within one month of LT.

**Conclusions:**

In our cohort, the incidence of new-onset AAs was 32% after bilateral LT. AA patients experienced worse short- and mid-term outcomes compared to controls. Furthermore, this study highlights older age and postoperative dobutamine administration as significant predictors of new-onset AA following bilateral LT. Further research is needed to clarify the causal relationships and long-term implications of AA on the clinical course of LT recipients.

## 1 Introduction

Lung transplantation (LT) is the established treatment for end-stage chronic respiratory failure not amenable to other medical or surgical therapies (1). Several clinical conditions require LT, including cystic fibrosis, chronic obstructive pulmonary disease, and interstitial lung disease. LT recipients represent a special and growing population. Advances in surgical techniques and perioperative care have significantly improved the one-year survival rate for LT, increasing from 73% in the 1990s to approximately 84% today (2). Furthermore, the number of LT recipients has doubled since 2000, with approximately 2,000–2,500 single or bilateral lung transplants performed annually in the United States and over 4,000 worldwide, yielding an estimated five-year survival rate of 50% (1–4).

Among the postoperative complications of LT, arrhythmias emerge as a significant risk factor associated with negative prognostic implications; however, few studies and limited data are available on this topic (5–8). Atrial arrhythmias (AA), including atrial fibrillation (AF), atrial flutter (AFL), and atrial tachycardia (AT), are the most commonly observed arrhythmias following LT (6). These arrhythmias occur in approximately 19%–46% of LT patients during the immediate postoperative period and in about 14% during long-term follow-up (5, 6). Specifically, postoperative AF is defined as a new-onset cardiac arrhythmia arising in the immediate postoperative period, with an incidence ranging from 2% to 30% and peaking between postoperative days 2–4 (9). Postoperative AF is associated with an increased risk of stroke, myocardial infarction, and death compared to AF of non-postoperative origin (9).

It is important to distinguish between early postoperative atrial tachyarrhythmias, which occur before hospital discharge or within three months post-LT, and late atrial tachyarrhythmias, occurring more than 3–6 months post-LT (6).

Indeed, our observational study aims to assess: (i) the incidence of new-onset postoperative AAs within one month of bilateral LT; (ii) their impact on short- and mid-term outcomes; and identifying (iii) potential predictors.

## 2 Materials and methods

**Figure 1:**
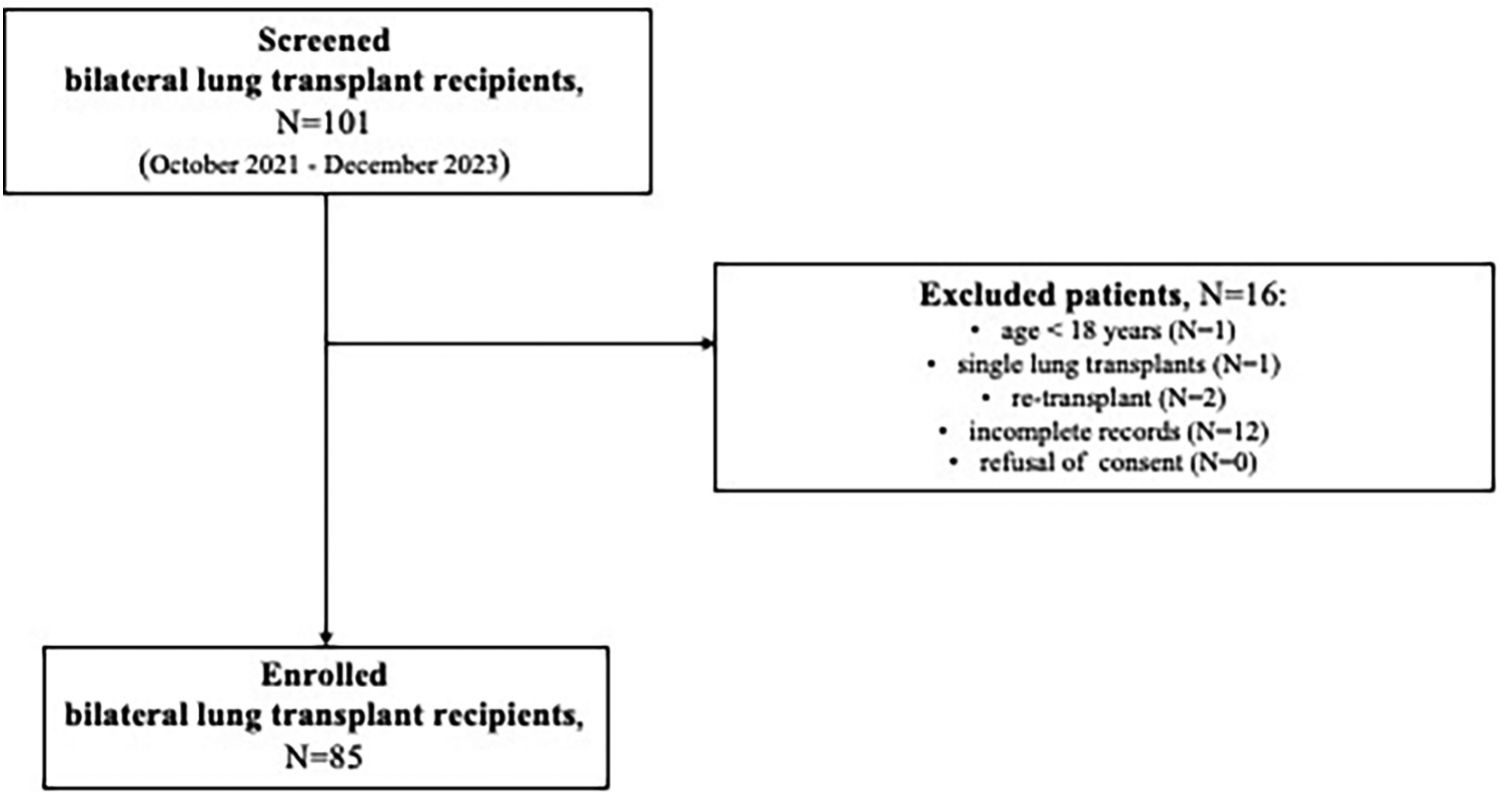
Study flowchart.

The study was approved by the local Institutional Ethic Committee (reference 4539_AO_18) and conducted in accordance with the principles of Good Clinical Practice outlined in the Declaration of Helsinki. The informed consent was obtained from all participants. This article was written in accordance with the STROBE checklist (Supplementary Table S1) (10). All consecutive patients, undergoing to the first bilateral LT at Padua University Hospital, were retrospectively screened between October 2021 and December 2023. Predefined exclusion criteria were: (1) age < 18 years old; (2) single transplant; (3) re-transplant; (4) incomplete records (i.e., missing echocardiographic data and/or outcomes of interest); and (5) refusal of consent. The study flowchart is depicted in Figure 1.

The following variables were collected from electronic health records (Tables 1–3): *(i)* demographic data [age, gender, body mass index (BMI)]; *(ii)* comorbidities; *(iii)* therapies at home (i.e., corticosteroids or O_2_-therapy); *(iv)* underlying diseases leading to LT (septic, interstitial, obstructive or others) (*see full description in* Table 1); *(v)* preoperative cardiac measurements (including parameters derived from right heart catheterization (11, 12) and ecographic measurements of cardiac function (11, 13) (*see full description in* Table 2); *(vi)* intra-operative characteristics and management; *(vii)* hemodynamic characteristics after ICU admission [i.e., pulse contour cardiac output (PICCO) parameters, vasoactive and inotropic support]; *(viii)* incidence of arrhythmic events and treatments (*see full description in* Table 3); and then *(xi)* outcomes of interest (*see full description in* Table 4).

**Table 1:** Baseline characteristics of bilateral lung transplant recipients.

	Overall *N* = 85 (100)	Arrhythmia group *N* = 27 (32)*	Control group *N* = 58 (68)	*P-value*
Demographic characteristics				
Age, years	56 [41.25–63]	61 [54.75–64.25]	51 [39.5–61]	***0***.***002***
Female gender, *n* (%)	57 (66)	21 (77.8)	36 (62)	0.15
BMI, kg/m^2^	24.1[19.6–26.5]	24.8 [22.2–27.4]	23.25 [18.8–26.37]	0.16
Comorbidities				
Hypertension, *n* (%)	31 (36)	13 (48.1)	18 (31)	0.13
Obesity, *n* (%)	3 (3,5)	0 (0)	3 (5.2)	0.23
Diabetes, *n* (%)	13 (15)	6 (22.2)	7 (12)	0.24
Hyperlipidemia, *n* (%)	10 (11,6)	5 (18.5)	5 (8.6)	0.19
Coronary heart disease, *n* (%)	8 (9,3)	5 (18.5)	3 (5.2)	***0***.***050***
Chronic renal disease, *n* (%)	0 (0)	0 (0)	1 (1.7)	1.00
Arrhythmic disease, *n* (%)	3 (3,5)	1 (3.7)	2 (3.4)	0.95
Therapies at home				
O_2_ therapy at rest, L/min	2.0 [1.0–4.0]	2.0 [0–3.0]	2.0 [1.0–4.0]	0.29
O_2_ therapy during activity, L/min	2.0 [0–5.0]	3.0 [0–5.0]	0.5 [0–5.0]	0.12
Underlying diseases				
Septic^a^, *n* (%)	13 (15.3)	3 (11.2)	10 (17.2)	0.53
Interstitial^b^, *n* (%)	30 (35.3)	5 (18.5)	25 (43.1)	0.32
Obstructive^c^, *n* (%)	31 (36.4)	15 (55.5)	16 (27.7)	***0***.***004***
Others^d^, *n* (%)	11 (13)	4 (14.8)	7 (12)	0.45
Preoperative characteristics				
Mechanical ventilation, *n* (%)	2 (2,3)	1 (3.7)	1 (1.7)	0.57
V-V ECMO^a^, *n* (%)	4 (4,7)	1 (3.7)	3 (5.2)	0.76
Pre-existing bacterial isolation, *n* (%)	10 (11,6)	2 (7.4)	8 (13.8)	0.40
Hemoglobin, g/dl	13.9 [12.7–14.7]	14.2 [12.42–15.12]	13.9 [12.7–14.7]	0.64
White blood count, cells 10^−9^/L	1.52 [1.16–2.6]	1.59 [1.17–2.73]	1.5 [1.14–2.60]	0.68

* More than 80% of arrhythmia occurred within the first week after lung transplant.

^a^ Septic: cystic fibrosis, bronchiectasis.

^b^ Interstitial: idiopathic pulmonary fibrosis, allergic extrinsic alveolitis, non-specific interstitial pneumonia, fibrosing emphysema, lymphocytic interstitial pneumonia, respiratory bronchiolitis interstitial lung.

^c^ Obstructive: chronic obstructive pulmonary disease, emphysema.

^d^ Others: idiopathic pulmonary hypertension, veno-occlusive disease, connective tissue disease, α1-anti-tripsin deficiency, lymphangioleiomyomatosis, histiocytosis, sarcoidosis, graft vs. host disease.

Table 1 Data are expressed as number and (percentage) or median and [interquartile range].

BMI, body mass index; V-V ECMO, venovenous extracorporeal membrane oxygenation.

The bold with italics values identified the significant data.

**Table 2:** Preoperative cardiac measurements.

	Overall *N* = 85 (100)	Arrhythmia group *N* = 27 (32)	Control group *N* = 58 (68)	*P-value*
Right heart catheterization				
mPAP, mmHg	19.5 [16.25–24]	18.5 [16.5–24]	20 [16–24.25]	0.84
PCWP, mmHg	9 [6.25–12]	10 [7.5–12]	8.5 [6–12]	0.61
CI, L/min	3.04 [2.76–3.58]	2.88 [2.50–3.49]	3.14 [2.84–3.59]	0.31
mRAP, mmHg	3.8 [2.77–5.23]	3.8 [2.72–5.03]	3.8 [2.83–5.44]	0.36
Echocardiographic measurements				
LV-EDV, ml	79.5 [69.25–98]	82.0 [75.0–100.5]	78 [66–95]	0.30
FE, %	60 [57–63]	61.0 [58.0–64.0]	59.5 [56–61.75]	0.10
Mitral regurgitation (reference: yes)	30 (3.52)	13 (48.1)	17 (29.3)	0.07
*mild*	30 (35)	13 (48.1)	17 (29.3)	
*moderate*	0 (0)	0 (0)	0 (0)	
Tricuspid regurgitation (reference: yes)	45 (52.9)	16 (59.3)	29 (50)	0.13
*mild*	42 (49)	14 (51.9)	28 (48.3)	
*moderate*	3 (4)	2 (7.4)	1 (1.7)	
Pulmonary regurgitation (reference: yes)	27 (31.8)	9 (33.3)	18 (31)	0.81
*mild*	26 (31)	9 (33.3)	17 (29.3)	
*moderate*	1 (1)	0 (0)	1 (1.7)	
E/A ratio	0.87 [0.72–1.11]	0.79 [0.70–1.22]	0.96 [0.77–1.23]	0.31
FAC, %	38.5 [35–43]	39 [36–42]	38 [35–45]	0.96
TAPSE, cm	1.99 [1.79–2.30]	1.97 [1.65–2.29]	2.0 [1.82–2.34]	0.30
sPAP, mmHg	31 [26–40.75]	36 [27–42.75]	30 [25.25–39.75]	0.20

Table 2 data are expressed as number and (percentage) or median and [interquartile range].

mPAP, mean pulmonary artery pressure; PCWP, pulmonary capillary wedge pressure; CI, cardiac index; mRAP, right atrial pressure; LV-EDV, left-ventricular end-diastolic volume; FE, ejection fraction; E/A ratio, refers to the ratio of the early (E) to late (A) ventricular filling velocities; FAC, fractional area change; TAPSE, tricuspid annular plane systolic excursion; sPAP, pulmonary artery systolic pressure.

**Table 3:** Characteristics of bilateral lung transplant recipients.

	Overall *N* = 85 (100)	Arrhythmia group *N* = 27 (32)	Control group *N* = 58 (68)	*P-value*
Intraoperative characteristics and management				
Time of LT, minutes	360 [311.25–400]	367 [307.5–422.25]	360 [318.75–400]	0.80
Time of ECMO, minutes	190 [163–225]	192.5 [162.25–276.25]	190 [163.5–218]	0.20
VIS^56.^	500 [212.5–1,501]	405 [276.25–1529.5]	502 [205–1,500]	0.96
Dobutamine*, mcg/kg/min	0 [0–3]	2.50 [0.0–5.0]	0 [0–3]	0.97
Norepinephrine*, mcg/kg/min	0.15 [0.015–0.2]	0.15 [0.03–0.25]	0.13 [0–0.2]	0.51
Nitric oxide, ppm	20 [20–30]	22.5 [20–30]	20 [20–30]	0.25
Blood transfusion, units	2.5 [2–4]	2 [1.5–4]	3 [2–4]	0.45
Plasma, units	0 [0–0.75]	0 [0–1]	0 [0–0]	0.08
Platelet, units	0 [0–0]	0 [0–0]	0 [0–0]	0.06
Fibrinogen, g	2 [2–3–25]	2.5 [2–4.25]	2 [2–3]	0.15
Fluid balance, ml	1,870 [1,100–2,600]	1,860 [1,115–2,785]	1,900 [1,050–2,500]	0.65
After ICU admission				
MAP, mmHg	76 [71–82]	78 [70.75–83.75]	76 [71–81.25]	0.91
Heart rate, mmHg	92 [82.25–100–5]	94 [86.25–104.25]	90 [79.75–99.5]	0.33
mPAP, mmHg	17.2 [14–21]	19.0 [14.0–21.0]	17 [14–20.75]	0.91
PICCO parameters				
CI, ml/min	2.44 [1.86–2.78]	2.49 [1.97–2.97]	2.42 [1.78–2.72]	0.73
GEF, %	19.5 [16.25–23.75]	19 [16–23.5]	19.5 [17.25–24.5]	0.57
ELWI, ml/kg	10.15 [8.50–11.6]	11.5 [6.3–16.0]	10.10 [8.8–10.7]	0.30
SVRI, dyn*s*cm−5*m^2^	2,392 [2,059–3,206]	2,309 [1679.0–2936.0]	2,567 [2,124–3,316]	0.20
Pulse pressure variation, *n* (%)	16.5 [10–22]	17.5 [10.75–23.25]	16.5 [10–20.75]	0.66
Stroke volume variation, *n* (%)	16.5 [13.5–27.25]	16 [15–27]	17 [12–28]	0.92
Vasoactive and inotropic agents				
Dobutamine*, mcg/kg/min	2.6 [0–3.6]	3 [2.2–5]	1.95 [0.0–3.0]	***0*.*017***
Dobutamine, duration	0.0 [1.0–2.0]	2 [1–3]	0.75 [0–2]	***0*.*039***
Norepinephrine*, mcg/kg/min	0.2 [0.11–0.3]	0.24 [0.14–0.30]	0.18 [0.11–0.30]	0.57
Norepinephrine, duration (days)	3.0 [2.0–4.0]	4 [2–5]	2 [2–4]	***0*.*009***
Dopamine*, mcg/kg/min	0 [0–0]	0 [0–0]	0 [0–0]	0.72
Dopamine, duration (days)	0 [0–0]	0 [0–0]	0 [0–0]	0.49
Epinephrine*, mcg/kg/min	0 [0–0]	0 [0–0]	0 [0–0]	0.30
Epinephrine, duration (days)	0 [0–0]	0 [0–0.13]	0 [0–0]	0.63

* Maximum dosage of amines.

Table 3 data are expressed as number and (percentage) or median and [interquartile range].

LT, lung transplantation; ECMO, extracorporeal membrane oxygenation; VIS, vasoactive inotropic score; CI, cardiac index; GEF, global ejection fraction; ELWI^f^, extravascular lung-water index; SRI, systemic vascular resistance index; MAP, mean arterial pressure; mPAP, mean pulmonary artery pressure; PICCO, pulse contour cardiac output.

The bold with italics values identified the significant data.

**Table 4:** Outcomes of bilateral lung transplant recipients.

	Overall *N* = 85 (100)	Arrhythmia group *N* = 27 (32)	Control group *N* = 58 (68)	*P-value*
PGD° at 48 h, *n* (%)	6 (6)	3 (11)	3 (5)	0.377
PGD° at 72 h, *n* (%)	4 (4)	2 (7)	2 (3)	0.589
28-day VFD, (days)	26 (24–27)	24 [21–26]	26 [25–27]	0.10
IMV, duration (hours)	41 (24–69.75)	69.5 [24–134.5]	36 (22.5–48)	***0***.***022***
n-IMV, *n* (%)	70 (81.4)	21 (77.8)	49 (84.5)	0.25
n-IMV, duration (hours)	11 (5–22)	18.5 [4.5–27]	9.5 (5–18)	0.89
Pronation, *n* (%)	17 (19.8)	9 (33.3)	8 (13.8)	***0***.***037***
Tracheostomy, *n* (%)	13 (15)	8 (29.6)	5 (8.6)	***0***.***013***
IVAC^57^, *n* (%)	19 (22)	11 (40.7)	8 (13.8)	***0***.***004***
Postoperative V-A ECMO, *n* (%)	12 (14)	8 (29.6)	4 (6.9)	***0***.***004***
Postoperative V-A ECMO, duration (days)	0 (0–0)	0 (0–3)	0 (0–0)	***0***.***005***
Postoperative V-V ECMO, *n* (%)	2 (2.3)	1 (3.7)	1 (1.7)	0.56
Postoperative V-V ECMO, duration (days)	0 (0–0)	0 (0–0)	0 (0–0)	0.45
Postoperative bleeding, *n* (%)	9 (10.5)	3 (11.1)	6 (10.3)	0.93
Anastomotic complications, *n* (%	15 (17.4)	8 (29.6)	7 (12.1)	***0***.***050***
Renal replacement therapy, *n* (%)	6 (7)	3 (11.1)	3 (5.3)	0.33
ICU LOS, (days)	6 (4–8)	8 (6.0–14.0)	5 (4–6)	***0***.***008***
Hospital LOS, (days)	31 (28–37.5)	33 (29.75–48.75)	31 (27–36)	0.12
ICU mortality, *n* (%)	5 (5.8)	1 (3.7)	4 (6.9)	0.55
Hospital Mortality, *N* (%)	2 (2.3)	1 (3.7)	1 (1.7)	0.59

Table 4 data are expressed as number and (percentage) or median and [interquartile range].

PGD at 48 h, primary graft dysfunction = 3 at 48 h from ICU admission; PGD at 72 h, primary graft dysfunction = 3 at 72 h from ICU admission; VFD, ventilator-free days; IMV, invasive mechanical ventilation; n-IMV, non-invasive mechanical ventilation; V-A ECMO, venoarterial extracorporeal membrane oxygenation; V-V ECMO, venovenous extra-corporeal membrane oxygenation; IVAC, infection-related ventilator-associated complications; ICU, intensive care unit; LOS, length of stay.

The bold with italics values identified the significant data.

Specifically, all right heart catheterizations and echocardiographic examinations were performed within routine clinical practice by multiple trained cardiologists and sonographers using diverse commercially available machines and then, patients' measurements were reviewed by expert cardiologists (PMM and CD). All echocardiographic measurements were evaluated and graded according to international guidelines (9, 14–16).

All transplants were performed with a central V-A ECMO support (17–19). Intraoperative inotropic and vasoactive support was administered according to the clinical judgement of the anesthesiologist in charge on the basis of advanced hemodynamic monitoring, including transpulmonary thermodilution, pulmonary artery catheter, and transesophageal echocardiography (20). Anesthetic management, immunosuppressive treatments and ventilations protocols were standardized in our institution, since 2021, consistently with international recommendations (18, 21–26). The 28-day ventilator free days were defined as the number of days of unassisted breathing to day 28 without having to reinstitute invasive ventilation. Patients who died before day 28 were assigned 0 (27). The follow-up is continued up to 3 months after surgery.

### 2.1 Statistical analysis

Continuous data were presented as mean and standard deviation (SD) when normally distributed or as the median with interquartile range [IQR] when non-normally distributed. Categorical data were summarized using absolute and (relative frequencies). Comparison of two groups of categorical variables were performed using Student's *t*-test of unpaired samples, while in case of non-normality or small sample size, Mann–Whitney *U* test was used. No imputation for missing data has been planned.

A multiple univariable logistic regression model was used to identify independent risk factors for AA. In multivariable logistic regression analysis we use significant variables from the univariate analysis that recorded a *p*-value < 0.1 (Supplementary Table S2). Moreover, the univariable analysis was applied to all clinical variables shown in Tables 1–3. Models were checked for collinearity and variables with Variance Inflation Factor (VIF) greater than 4 were excluded. All statistical tests were 2-tailed, and statistical significance was defined as *p* < 0.05. All analyses have been conducted using R version 4.0.3 (R foundation for Statistical Computing, Vienna, Austria).

## 3 Results

### 3.1 Baseline clinical characteristics

This study screened 101 LTs and finally analyzed 85, divided into two groups: the AA Group (27 patients, 32%) and the control Group (58 patients, 68%). Sixteen patients were excluded for the following reasons: age < 18 years (n. 1), single lung transplants (n. 1), re-transplant (n. 2), incomplete records (n. 12), refusal of consent (n. 0) (Figure 1).

Among patients in the AA group, AF was the most common arrhythmia (15, 55.6%), followed by paroxysmal supraventricular tachycardia (8, 29.6%) and mixed arrhythmias (4, 14.8%). Most patients (22, 81.5%) received either antiarrhythmic drugs or electrical cardioversion, while four subjects (14.8%) required a combination of both. One patient (3.7%) did not receive any treatment. Amiodarone was the primary antiarrhythmic used (26, 96%). Of those, 4 (15%) patients were also treated with beta-blockers.

With regards to baseline characteristics, patients in the AA Group were significantly older (median 61 years) compared to the control Group (median 51 years, *p*-value 0.002) (Table 1). Coronary heart diseases were more frequent in the AA Group (18.5% vs. 5.2%, *p*-value 0.05), while all other comorbidities were comparable between groups. According to end-stage underlying diseases, obstructive lung disease (e.g., COPD, emphysema) was significantly more common in the AA Group (55.5%) than in the control Group (27.7%) (*p*-value 0.004) (Table 1).

As shown in Tables 2, 3, there were no significant differences in preoperative cardiac measurements, intraoperative characteristics, blood transfusion requirements (including red blood cells, plasma, platelets, and fibrinogen) between groups (Table 3). Similarly, after ICU admission, mean arterial pressure, heart rate, mean pulmonary artery pressure, PICCO parameters (including cardiac index, global ejection fraction, extravascular lung-water index, systemic vascular resistance index, pulse pressure variation, and stroke volume variation) did not differ significantly (Table 3).

Interestingly, AA-patients required higher doses (*p*-value 0.017) and more prolonged infusions of dobutamine (*p*-value 0.039), as compared to controls. Similarly, norepinephrine duration was significantly longer in the AA group than in the control group (4 vs. 2 days, *p*-value 0.009) (Table 3).

### 3.2 Secondary outcomes

The incidence of primary graft dysfunction (PGD) at both 48- and 72 h post-LT was similar between the AA and control groups (81.5% vs. 77.6% at 48 h, *p*-value 0.10; 81.5% vs. 75.8% at 72 h, *p*-value 0.55). However, patients in the AA group required significantly longer invasive mechanical ventilation (IMV) compared to controls (69.5 vs. 36 h, *p*-value 0.022) (Table 4). Patients in the AA group had a significantly higher rate of pronation therapy (33.3% vs. 13.8%, *p*-value 0.037) and tracheostomes (29.6% vs. 8.6%, *p*-value 0.013), indicating a more complex postoperative respiratory course (Table 4). The need for ECMO and its postoperative duration were greater in the AA group compared to the control group (29.6% vs. 6.9%, *p*-value 0.004), with a longer ECMO duration (*p*-value 0.005) (Table 4). Patients in the AA group had a significantly higher incidence of infection-related ventilator-associated complications (40.7% vs. 13.8%, *p*-value 0.004) and anastomotic complications (29.6% vs. 12.1%, *p*-value 0.050) (Table 4). Finally, the ICU length of stay (LOS) was significantly longer in the AA group compared to the control group (8 vs. 5 days, *p*-value 0.008) (Table 4).

### 3.3 Risk factors for developing Aa

The multivariate results indicate that only age (OR 1.11, 95% CI 1.01–1.25, *p*-value of 0.047) (Table 5) and a high postoperative dobutamine dosage (OR 2.25, 95% CI 1.15–5.01, *p*-value 0.026) were significant predictors of postoperative new-onset AA (Table 5).

**Table 5:** Logistic regressions.

Predictors	Univariable			Multivariable		
	OR	95% CI	*P- value*	OR	95% CI	*P-value*
Baseline characteristics						
Age, year	1.07	1.03–1.13	0.005	1.11	1.01–1.25	***0***.***047***
Coronary heart disease, *n* (%)	4.17	0.94–21	0.065	3.62	0.38–39	0.262
Preoperative echocardiographic measurements						
Mitral regurgitation (reference: yes)%	3.06	0.95–11	0.072	3.31	0.59–25	0.194
Intraoperative characteristics and management						
Dobutamine*, mcg/kg/min	1.20	0.97–1.50	0.087	1.16	0.72–1.93	0.538
Postoperative vasoactive and inotropic agents						
Dobutamine*, mcg/kg/min	1.33	1.05–1.73	0.022	2.25	1.15–5.01	***0***.***026***
Dobutamine, duration	1.36	1.01–1.91	0.051	0.53	0.20–1.18	0.146
Norepinephrine, duration (days)	1.26	1.05–1.59	0.029	1.47	0.99–2.57	0.128

* Maximum dosage of amines.

Table 5 data are expressed as odds ratio (OR) and confidential interval (CI).

The bold with italics values identified the significant data.

Finally, at 3 months postoperatively, there were no new cases of AA. Only one patient (3.7%) remained on oral anticoagulation, while six patients (22.2%) were still taking antiarrhythmic drugs.

## 4 Discussion

This single center observational study, conducted in a homogeneous cohort of bilateral LT, aims to assess the incidence and predictors of new-onset AA after LT, as well as the potential impact of postoperative AA on patients' outcomes.

The results demonstrate that the AA Group had significant differences in baseline characteristics, including older age and a higher prevalence of coronary heart disease and obstructive lung disease. These findings suggest that patients in the AA Group had more comorbidities, which could potentially contribute to the higher incidence of AA post-LT. In fact, the etiology of postoperative AA is multifactorial, involving surgical manipulation (28), ischemia-reperfusion injury (29), electrolyte imbalances (30), and heightened sympathetic activity and comorbidities, including smoking, hypertension (31), and diabetes mellitus (32).

Hypertension, and diabetes mellitus are well-established comorbidities associated with up to a two-fold increased risk of AF, inducing oxidative stress, inflammation, atrial fibrosis (33), and promoting structural and electrical remodeling of the atrium (31, 32, 34, 35).

The study found that AF was the most common arrhythmia in the AA Group, which is consistent with previous literature that reports a higher incidence of AAs in post-lung LT patients (6, 28). Many of these patients were treated with antiarrhythmic drugs, primarily amiodarone, highlighting the importance of managing arrhythmias in this cohort (36–38). Notably, no new cases of AF were observed at 3 months after LT, and most patients had discontinued antiarrhythmic drugs by this time, suggesting that arrhythmias may resolve or become manageable with appropriate treatment, as recently published (28, 39).

The occurrence of AA seems to contribute to a more complicated respiratory recovery and a longer need for postoperative V-A ECMO could be related to a higher postoperative cardiovascular instability associated with arrhythmias. Moreover, patients in the AA Group had a significantly higher incidence of IVAC and anastomotic complications. This may suggest that arrhythmias contribute to a more fragile postoperative state, increasing susceptibility to infections and other complications.

Keeping in line, the occurrence of AA after noncardiac thoracic surgery in the elderly population strongly correlates with worse outcomes, such as increased ICU LOS and mortality (40–42). Nowadays, neither effective screening strategies nor targeted prophylaxis have been tested, therefore, a close monitoring could be recommended to the higher risk patients.

The multivariate analysis revealed that age and postoperative dobutamine administration were significant predictors of postoperative AA. This finding emphasizes the role of age as a risk factor for AA, as older patients may have a lower cardiovascular reserve, making them more prone to arrhythmic events. Likewise, previous data in literature reported age older than 50 years as an independent risk factor for higher prevalence of AA and more severe arrhythmia type (43), suggesting for each unit increased in age, a greater odds to develop AA of 4.5% (44). This multifactorial relationship has not yet been well understood, although the disruption of the intracellular calcium regulation system, the scars and the effects of other chronic pathologies have been held countable (45, 46). Additionally, also the use of dobutamine, a β1-adrenergic agonist commonly used to enhance cardiac output in patients with low cardiac function, was identified as a key predictor of new-onset AA, suggesting that the use of dobutamine may be both a consequence and a contributing factor to arrhythmias after LT. However, its stimulatory effect on the myocardium can predispose patients to AA, particularly AF (47–49).

Despite the significant findings, several limitations should be considered. The sample size was relatively small, and the study was an observational, retrospective and single-center investigation, meaning causality cannot be definitively established. Additionally, while the study provides valuable insights into the relationship between AA and postoperative complications, other unmeasured variables, such as genetic predisposition, may also contribute to the observed outcomes. Further research is needed to elucidate the causal relationships and long-term implications of AA on the clinical course of LT recipients.

In conclusion, this study, showing an incidence of new-onset AA after LT of 32%, highlights that age and postoperative dobutamine administration are significant predictors of new-onset AA following bilateral LT. Moreover, AA patients experienced worse short- and mid-term outcomes (higher rate of pronation therapy, tracheostomy, complex postoperative respiratory course, greater rate of prolonged V-A ECMO, IVAC, anastomotic complications and longer ICU LOS). Indeed, clinicians should be aware that new-onset atrial arrhythmias are common after bilateral lung transplantation, particularly in older patients and those receiving postoperative dobutamine. These arrhythmias are associated with more complex respiratory recovery, a higher risk of postoperative complications, and longer ICU stays. Early identification and close monitoring of high-risk patients are essential to improve outcomes and guide timely management of arrhythmias in this setting.

## Data Availability

The raw data supporting the conclusions of this article will be made available by the authors, without undue reservation.
